# Association between intratumoral lymphatic microvessel density (LMVD) and clinicopathologic features in endometrial cancer: a retrospective cohort study

**DOI:** 10.1186/1477-7819-8-89

**Published:** 2010-10-14

**Authors:** Lecy Kawamura, Filomena M Carvalho, Bernardo GL Alves, Carlos E Bacchi, Joao Carlos Sampaio Goes, Marcelo Alvarenga Calil, Edmund C Baracat, Jesus P Carvalho

**Affiliations:** 1 Department of Obstetrics and Gynaecology of Faculdade de Medicina da Universidade de São Paulo, Sao Paulo (SP), Brazil; 2 Department of Pathology of Faculdade de Medicina da Universidade de São Paulo, Sao Paulo (SP), Brazil; 3 Consultoria em Patologia, Botucatu (SP), Brazil; 4 Instituto Brasileiro de Controle do Cancer, Sao Paulo (SP), Brazil

## Abstract

**Background:**

Lymph node metastasis in endometrial cancer significantly decreases survival rate. Few data on the influence of intratumoral lymphatic microvessel density (LMVD) on survival in endometrial cancer are available. Our aim was to assess the intratumoral LMVD of endometrial carcinomas and to investigate its association with classical pathological factors, lymph node metastasis and survival.

**Methods:**

Fifty-seven patients with endometrial carcinoma diagnosed between 2000 and 2008 underwent complete surgical staging and evaluation of intratumoral LMVD and other histologic variables. Lymphatic microvessels were identified by immunohistochemical staining using monoclonal antibody against human podoplanin (clone D2-40) and evaluated by counting the number of immunostained lymphatic vessels in 10 hot spot areas at 400× magnification. The LMVD was expressed by the mean number of vessels in these 10 hot spot microscopic fields. We next investigated the association of LMVD with the clinicopathologic findings and prognosis.

**Results:**

The mean number of lymphatic vessels counted in all cases ranged between 0 and 4.7. The median value of mean LMVD was 0.5, and defined the cut-off for low and high LMVD. We identified low intratumoral LMVD in 27 (47.4%) patients and high LMVD in 30 (52.6%) patients. High intratumoral LMVD was associated with lesser miometrial and adnaexal infiltration, lesser cervical and peritoneal involvement, and fewer fatal cases. Although there was lower lymph node involvement among cases with high LMVD, the difference did not reach significance. No association was seen between LMVD and FIGO staging, histological type, or vascular invasion. On the other hand, low intratumoral LMVD was associated with poor outcome. Seventy-five percent of deaths occurred in patients with low intratumoral LMVD.

**Conclusion:**

Our results show association of high intratumoral LMVD with features related to more localized disease and better outcome. We discuss the role of lymphangiogenesis as an early event in the endometrial carcinogenesis.

## Background

Endometrial cancer is the most frequent gynecologic malignancy in the western world. The death rate has increased during the most recent decades, probably due to an increase in life span and coexisting medical comorbidities [1]. The most important prognostic factors in endometrial cancer are histological type, grade, lymph node status, deep myometrial invasion, and stage [2,3]. Approximately 72% of endometrial cancers are stage I, 12% stage II, 13% stage III, and 3% stage IV ^(1)^. Five year survival is about 90% in disease confined to the uterus, but drops to 60% when lymph nodes are positive [4]. Lymph node metastasis is a complex, multi-step biological process initialized by tumor cells, involving stroma invasion, new lymphatic vessel formation, and spread through lymphatic vessels to lymph node. Understanding how cancer cells gain access to lymphatic channels and develop metastases is of great interest for development of new therapeutic strategies targeted against members of the signaling pathways involved in the lymphangiogenesis process.

The growth of lymphatic vessels largely depends on many growth factors, such as vascular endothelial growth factor-C and -D (VEGF-C and VEGF-D), platelet derived growth factor-BB (PDGF-BB) and hepatocyte growth factor[5]. However, in cancer, these known lymphangiogenic factors lead to simultaneous stimulation of angiogenesis and lymphangiogenesis [6]. Lymphatic microvessel density (LMVD) is one of the ways to evaluate lymphangiogenesis. It was poor studied due to the difficulties associated with detecting and characterizing lymphatic markers. Currently, several molecules specifically expressed in lymphatic endothelial cells, such as podoplanin, LYVE1, and the homeobox gene *prox-*1, have enabled a more precise study of the lymphatic vasculature and the molecular mechanisms involved in lymphangiogenesis [7]. LMVD has been investigated in many tumors, particularly those characterized by lymphatic dissemination, such as cervical carcinomas [8-10], but there are few studies that have evaluated LMVD in endometrial carcinomas, and results have been conflicting [11,12].

Therefore, the aim of this retrospective study was to evaluate the relationship between intratumoral LMVD, as well as other histologic variables, and the incidence of lymph node metastasis and overall survival of patients with endometrial cancer following hysterectomy and lymph node dissection.

## Methods

### Patients

This study retrospectively analyzed patients with endometrial cancer treated at the Instituto Brasileiro de Controle do Cancer (IBCC), a reference Cancer Center in São Paulo, Brazil, between 2000 and 2008. This cohort of 57 patients included 28 (49.1%) with stage I disease, 4 (7.0%) with stage II disease, 22 (38.5%) with stage III disease, and 3 (5.2%) with stage IV disease, classified according to the 2009 revised International Federation of Gynecology and Obstetrics (FIGO) system [13]. Age of patients ranged from 46 to 89 years (67.4 ± 10.27 y). All patients had undergone surgical staging hysterectomy with a sample of pelvic lymph nodes. The number of lymph nodes excised ranged from 3 to 49 (21.5 ± 12.44), and they were positive in 22 (38.6%) and negative in 35 (61.4%) cases. Paraortic lymph nodes were assessed in 27 cases and nine of them were involved by the neoplasia. The follow-up ranged from 6 to 144 months (mean 39.54 ± 29.98 months). Twelve patients died of their disease between 6 and 108 months after the diagnosis (median 21.5 months). Data about myometrial, cervical, adnexal and peritoneal involvement, were retrieved from the medical records. This study was approved by the Department of Obstetrics and Gynecology Scientific Committee of the Faculdade de Medicina da Universidade de São Paulo, by the Ethical Committee for Research Projects of the Hospital das Clinical da Faculdade de Medicina da Universidade de São Paulo (Comissão de Ética para Análise de Pesquisa - CAPPesq) (process number 0728/08), and by the Ethical Committee for Research of the Instituto Brasileiro de Controle do Cancer.

### Tumor Specimens

Tumor slides were reviewed independently by two pathologists and were classified according to the criteria of the World Health Organization (WHO) Classification of Tumors [14]. The Vancouver system was applied to classify the tumors in low and high grades. A tumor was considered high grade if it showed at least two of these criteria: 1) predominantly papillary or solid growth pattern, 2) 6 or more mitotic figures/10 high power fields, or 3) severe nuclear atipia [15]. Peritumoral lymphovascular space invasion (LVSI) was also evaluated for all cases in the whole histological sections. A representative peripheral area of the tumor was selected for the construction of tissue microarray blocks and immunohistochemical study.

### Construction of tissue microarray (TMA) blocks

The tumor areas selected in the slides were marked in the corresponding paraffin donor blocks and one cylinder of material (2.0 mm in diameter) was punched from each case and mounted into recipient paraffin blocks at 1 mm intervals using a precision microarray instrument (Beecher Instruments, Silver Spring, MD). A grid system was established such that each core had an x and y coordinate reference for sample identification. Blocks were sealed at 60°C for 10 m. Three μm sections from each TMA block were then prepared using standard techniques and mounted on Starfrost^® ^slides. The first histological sections were stained by hematoxilin-eosin and examined to be sure the correct areas were included.

### Immunohistochemistry analysis

Histological sections (3 μm) from TMA blocks were quenched with 3% hydrogen peroxide solution in phosphate-buffered saline (PBS; Sigma, St. Louis, MO) for 20 m to block endogenous peroxidase activity. After several washes in PBS, sections were heated in a microwave (Electrolux, 900 W) for 15 m in 0.01 M citrate buffer, pH 6.0 for antigen retrieval, then cooled at RT for 20 m. Sections were then incubated overnight with a 1:400 dilution of monoclonal antibody against human podoplanin (clone D2-40, Dako, Carpinteria, CA). Podoplanin is a glycoprotein specifically expressed by lymphatic endothelial cells. In those tumor sections with negative podoplanin staining, adjacent normal-appearing lymphatic endothelial cells served as positive internal controls.

### Quantification of lymphatic microvessel density

**Figure 1:**
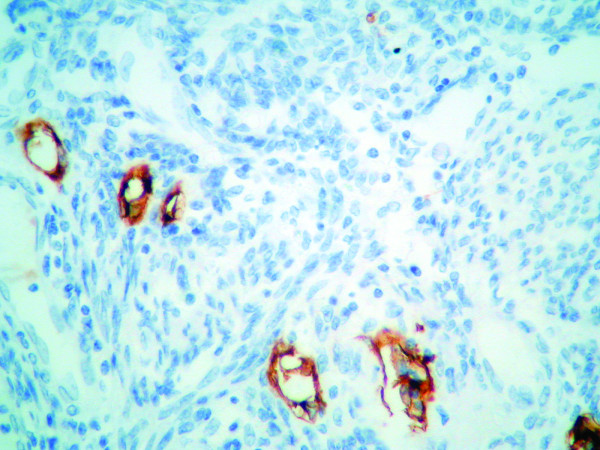
**Immunohistochemistry staining of a histological section of endometrioid carcinoma using D2-40 antibody against podoplanin showing six lymphatic vessels**. After counting 10 high power fields, the mean was calculated for each case. (Original magnification X400)

Stained TMA histologic sections were analyzed using standard light microscopy (Nikon, Eclipse 200). Under low magnification, the most vascularized intratumoral areas were identified. We counted the number of immunostained lymphatic vessels found in 10 hot spot areas at 400× magnification. Only vessels exhibiting typical morphology (lumen) were considered lymphatic microvessels (Figure 1). The LMVD for each case was expressed by the mean value (total number of vessels in 10 hot spot microscopic fields/10). The median value of all the mean LMVD was the cutoff to divide tumors in high or low LMVD, as suggested by Hall et al.[16].

### Statistical analyses

The association between categorical variables and lymph node status and survival were evaluated using Pearson's Chi-square test. Kaplan & Meier method was used to produce time life tables and survival curves.

A *p*-value less than 0.05 was considered significant. The software used was Epi Info^tm^, Version 3.5.1. [17].

## Results

**Figure 2:**
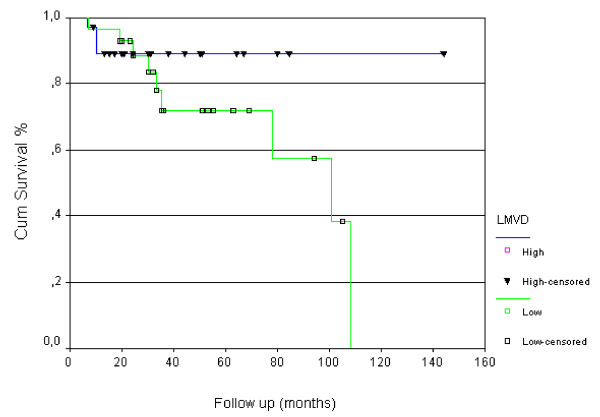
**Overall survival curve stratified for high and low intratumoral lymphatic microvessel density (LMVD)**.

The tumors were classified as endometrioid (44 cases, 77.2%) and non-endometrioid classified as serous (6 cases, 10.5%), clear cell (4 cases, 7%), undifferentiated (2 cases, 3.5%), and mixed (1 case, 1.7%). The association between all the variables with lymph node status and patient death are summarized in Table 1. The mean number of lymphatic vessels counted in each patient's tumor sample ranged from 0 to 4.7. The median value was 0.5 vessels and defined the value above and below which was considered high and low LMVD, respectively. Low LMVD was found in 27 (47.4%) patients, and high LMVD was found in 30 (52.6%) patients. We observed that low intratumoral LMVD was associated with poor outcome. Seventy-five percent of deaths occurred in patients with low intratumoral LMVD (p = 0.031). The mean survival time observed in the group with low LMDV was 79 months (confidence interval [62 - 96 months]) and the mean survival time for high LMDV group was 129 months (confidence interval [113 - 145 months]). The difference was significant at 5% level. The survival curves for both LMDV groups are presented at Figure 2.

**Table 1:** Clinicopathological variables and association with lymph node status and outcome

Variable	Categories	Lymph nodes		*p^1^*	Outcome		*p^1^*
		**negative** **n**	**positive** **n**		**alive** **n**	**dead** **n**	

Age	45-69 years	20	13	0.885	27	6	0.533
	≥70 years	15	9		18	6	
Histological type	Endometrioid	30	14	0.053	38	6	**0.010**
	Non-Endometrioid	5	8		7	6	
Histological grade (Vancouver system)	low	32	12	**0.001**	37	7	**0.080**
	high	3	10		8	5	
							
FIGO stage	I	27	1	**< 0.001**	24	4	0.303
	II	4	0		4	0	
	III	3	19		15	7	
	IV	1	2		2	1	
Vascular invasion	no	12	3	**0.017**	14	1	**0.040**
	yes	6	10		10	6	
							
LMVD^2^	Low	14	13	0.160	18	9	**0.031**
	high	21	9		27	3	
							
Myometrial infiltration	< 50%	25	5	**< 0.001**	26	4	0.113
	> 50%	9	17		18	8	
							
Cervical infiltration	no	25	9	**0.023**	31	3	**0.003**
	yes	9	12		12	9	
Adnaexal involvement	no	33	14	**0.023**	31	6	**0.004**
	yes	2	8		4	6	
							
Peritoneal involvement	no	28	13	**0.048**	33	8	0.811
	yes	1	5		4	2	

Total	57	34	22		45	12	

^1^*p *value (Chi-square of Pearson); ^2^Intratumoral lymphatic microvessel density

Low LMVD was also associated with miometrial and adnaexal infiltration, cervical and peritoneal involvement. Patients below 70 years presented higher LMVD (21/33, 63.3%) than older patients (9/24, 37.5%) although the difference did not reach statistical significance. No association was seen between LMVD and FIGO staging, histological type, vascular invasion, or lymph node involvement (Table 2).

**Table 2:** Association between intratumoral lymphatic microvessel density (LMVD) and clinicopathological features

Variable	categories	LMVD		p^1^
		**low** **n**	**high** **n**	

Age	45-69 years	12	21	0.051
	≥ 70 years	15	9	
				
Histological type	Endometrioid	19	25	0.396
	Non-Endometrioid	8	5	
Histological grade (Vancouver system)	low	20	24	0.594
	high	7	6	
				
FIGO stage	I	9	19	0.145
	II	2	2	
	III	14	8	
	IV	2	1	
				
Vascular invasion	no	6	9	0.576
	yes	8	8	
				
Myometrial infiltration	< 50%	10	20	0.034
	> 50%	17	9	
Cervical infiltration	no	12	22	**0.047**
	yes	14	7	
				
Adnaexal involvement	no	19	28	**0.023**
	yes	8	2	
				
Peritoneal involvement	no	17	24	**0.055**
	yes	5	1	
				
Lymph node involvement	no	14	21	0.160
	yes	13	9	
				
Outcome	alive	18	27	**0.031**
	death	9	3	

Total	57	27	30	

^1^*p *value (Chi-square of Pearson)

Positive lymph nodes were significantly associated with histological grade (p = 0.001), vascular invasion (p = 0.017), FIGO stage (p < 0.001), myometrial infiltration (p < 0,001), cervical infiltration (p = 0,023), adnaexal involvement (p = 0,023) and peritoneal involvement (p = 0,048). Besides LMVD, death was associated with histological type (p = 0.010), vascular invasion (p = 0.040), and lymph node involvement (p = 0.025). Mean survival time was 110 months (range of 84 - 135 months) for patients with negative lymph node involvement, and 69 months (range of 48 - 89 months) for patients with positive lymph nodes.

## Discussion

Lymphatic metastasis is one of the most important prognostic factors for survival of patients with endometrial cancer as well as for other epithelial malignancies. Many clinical and experimental data suggest that migration of tumor cells into the lymph nodes is facilitated by lymphangiogenesis[18,19]. Studies evaluating lymphatic neoformation and its role in tumoral migration are facilitated by the recent identification of lymphangiogenic factors and their receptors, and by lymphatic vascular markers [5].

The most commonly used markers for immunohistochemistry identification of lymphatic endothelial tissue include the vascular endothelial growth factor receptor 3 (VEGFR-3), PROX-1, LYVE-1, and podoplanin [5]. Among these, podoplanin, a 38 kDa mucin-type transmembrane glycoprotein which is recognized by the monoclonal antibody D2-40, is considered the most reliable marker with a high specificity and sensitivity [5]. In this study, we chose to evaluate podoplanin because it was found to be expressed in lymphatic, but not blood vascular endothelium [20], and is expressed in smaller lymphatic vessels rather than larger ones [20], allowing a more sensitive identification of lymphangiogenesis.

We then considered which compartment (intra or peritumoral) to evaluate for lymphangiogenesis in this study, as this is a source of great controversy in the literature. Since our knowledge of lymphangiogenesis in different types of cancer is still limited, we decided that it would be worthwhile to begin accumulating data. We believe that a better understanding of the role of lymphangiogenesis in tumoral dissemination will only be achieved after contributions from different researchers groups.

In this study, we evaluated LMVD in the intratumoral compartment using podoplanin as the lymphatic marker. Vascular density was determined by direct counting of vessels in 10 high power microscope fields. Although our study is limited by the fact that LMVD was assessed only in TMA sections, the area of the tumor that was investigated was rigorously chosen considering fixation conditions of the specimen, morphology of the tumor, and the peripheral localization of the sample. Besides, our cores had 2.0 mm in diameter, bigger than the conventional cores.

LMVD has been studied in several cancers by various methods [21]. There are few studies about LMVD in gynecological cancer. Gombos et al. [9] studied intra- and peritumoral LMVD using D2-40 immunohistochemistry in 111 cervical squamous cell carcinomas and correlated them with vascular endothelial growth factor (VEGF)-C expression, clinicopathologic tumor features, and outcome. They found only high peritumoral LMVD to be associated with poor overall survival. Zhang et al., using LYVE-1 (lymphatic vessel endothelial hyaluronan receptor-1) as the lymphatic marker, studied intra- and peritumoral lymphatic vascular density in early-stage invasive cervical carcinomas. Both intravascular and peritumoral high LMVD were associated with lymph node metastasis [8]. In a conflicting study, Birner et al. studied 95 cases of stage pTIb cervical carcinomas and observed a more favorable prognosis among tumors with high LMVD [10], a result that is similar to ours.

LMVD studies in endometrial cancer are extremely limited. To investigate whether increased peritumoral and intratumoral LMVD was a good prognostic factor for nodal metastasis, Gao et al. studied 102 patients with endometrial carcinoma using LYVE-1 as the lymphatic marker [22]. They found peritumoral but not intratumoral high LMVD to be associated with lymph node metastases. Vandenput et al. studied 62 patients with endometrial carcinoma and found that neither peritumoral nor intratumoral lymphangiogenesis determined by podoplanin expression were related to prognosis [11]. Donughue et al. evaluated lymphatic vessel density (LVD) in 17 cases of endometrial carcinomas by counting vessels expressing D2-40. Intratumoral LVD was significantly increased when compared with endometrial functionalis LVD, but not when compared with endometrial basalis LVD. They also observed a tendency towards increased intratumoral LVD in grade 3 tumors compared with grade 1 tumours [23]. Other studies have documented lymphatic metastases independent of intratumoral lymphatic vessels suggesting that the intratumoral lymphatic vasculature is usually not functioning and only peritumoral lymphatic vessels conduct fluid and cells [24].

In our study, low LMVD was associated with poor outcome. Seventy-five percent of deaths occurred in patients with low intratumoral LMVD compared with only 25% of deaths in patients with high LMVD (p = 0.031). In contrast, high LMVD was associated with favorable prognostic factors. High LMVD tumors were associated with less myometrial infiltration > 50% (31% *vs*. 63%, p = 0.034), less cervical involvement (24.1% *vs*. 53.8%, p = 0.023), and less peritoneal involvement (4% *vs*. 29.4%, p = 0.055). The explanation for this finding is not clear, but we can suppose that lymphangiogenesis is an early event in tumor progression, when lymphatic vessels are still not functional. Considering that higher LMVD was present in early anatomic tumors with no association with intrinsic tumors biological characteristics, as histological type or grade, we can suppose that this is an event related to the first steps of local interaction between tumor and microenvironment. Besides, high LMVD was associated with favorable outcome, indicating that it might be clinically relevant. However, it must be pointed that the vascular density results from a more complex biologic process that involves a cross-talk between tumor, vessels and host stroma. Our results showed higher LMVD among tumor in the initial steps of invasion (no extrauterine extension) and better prognosis. It is well known the role of normal lymphatic vasculature in maintaining tissue homeostasis [25]. The lymphatic network must be investigated not only by its anatomic density, as we did, but also functionally. We need intensive further investigation with more early cases and adequate follow up, to explore the role of vascular density in the tumoral progression. These future results may help elucidate pathways of lymphangiogenesis leading to identification of potential drug targets for endometrial carcinomas. Considering our preliminary results, it is possible that the target would not be the lymphatic vessel itself, but some other molecules involved with its function.

## Conclusions

Our results show that high intratumoral LMVD is a characteristic of endometrial carcinomas with lesser extrauterine extension and is associated with better outcome. However, further studies with larger series are needed to elucidate the biological meaning of these intriguing findings.

## Competing interests

The authors declare that they have no competing interests.

## Authors' contributions

LK collected the data and participated in pathological analyses of the samples and the drafting of the manuscript. FMC conceived of the study, participated in its design and review the slides, and helped to the coordination and drafting of the manuscript. BGLA reviewed all the histological samples and performed the immunohistochemical analyses. CEB carried out the immunohistochemical reactions and helped their interpretation. JCSP, MAC and ECB participated in the design and the coordination, and helped to draft the manuscript. JPC conceived of the study and participated in its design, coordination and drafting. All authors read and approved the final manuscript.
